# Melon-sized Parotid Pleomorphic Adenoma—A Case Report

**DOI:** 10.1007/s12070-021-02660-3

**Published:** 2021-05-28

**Authors:** Keerthan Dhanasekar, Heena Kapadia, Steven Neilaj, Meriam Abdeslem, Ganapathy Dhanasekar

**Affiliations:** 1 University of Sheffield Medical School, Beech Hill Rd, Broomhall, Sheffield, S10 2RX UK; 2 Scunthorpe General Hospital, Northern Lincolnshire and Goole NHS Foundation Trust, Cliff Gardens, Scunthorpe, North Lincolnshire, DN15 7BH UK

**Keywords:** Case report, Pleomorphic adenoma, Parotid, Management

## Abstract

This case report describes a 55 year-old gentleman who had been living with an extremely large (1.2 kg) pleomorphic adenoma for 24 years—despite its significant size, weight and appearance. A modified surgical incision and approach were required to remove the tumour, to avoid damaging the facial nerve and its branches.

## Introduction

Salivary gland tumours account for 8.1% of all head and neck tumours. There is a so-called rule of 80 s; 80% of all salivary tumours are of the parotid, 80% of parotid tumours are benign, and 80% of benign tumours that arise in the parotid are pleomorphic adenomas. In addition, pleomorphic adenomas of the submandibular and sublingual gland have been seen but are less common [1] This case report details the management of a patient with a large, long-standing pleomorphic adenoma of the parotid gland.

## Case Report

**Fig. 1:**
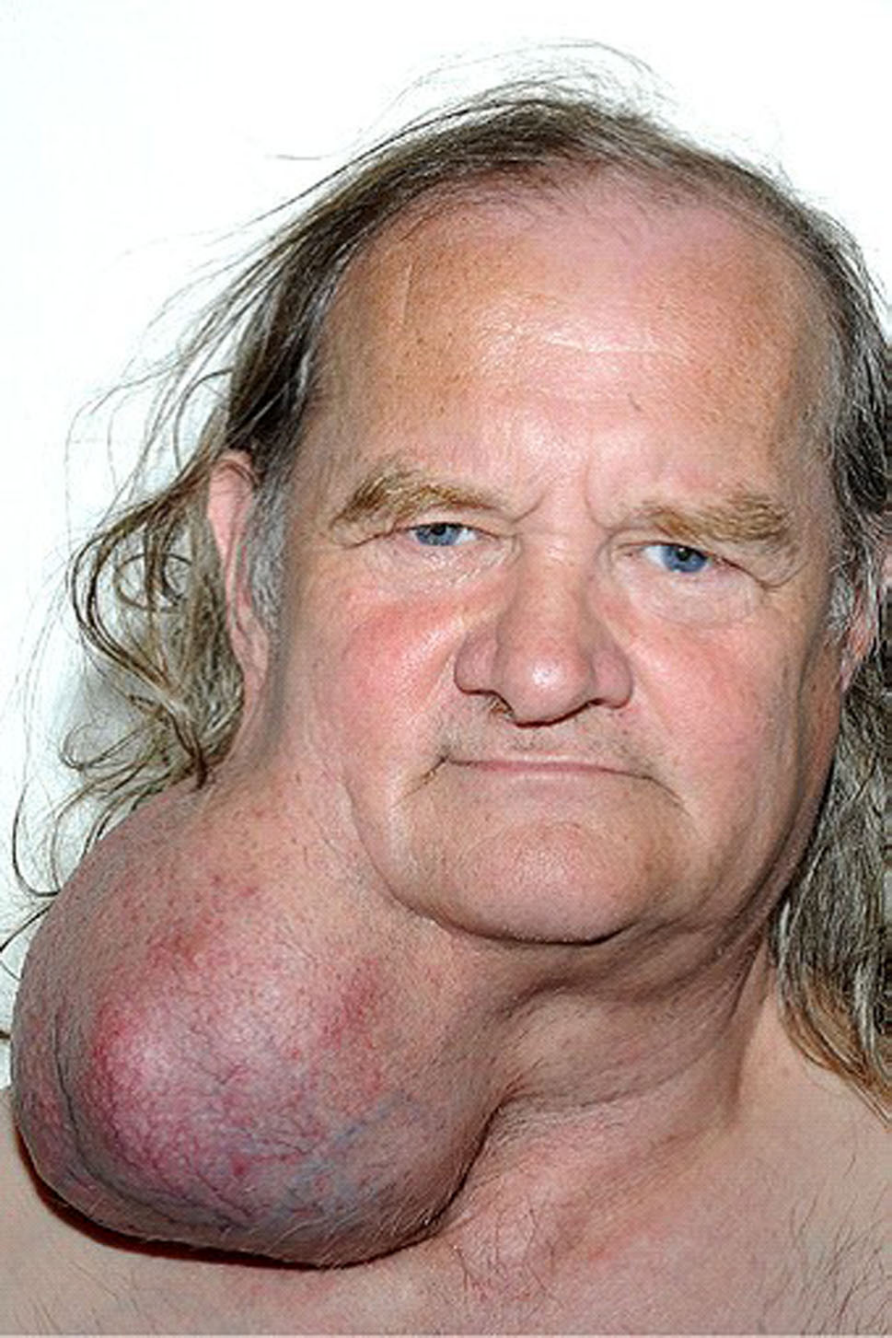
Photograph of patient, pre-operation

**Fig. 2:**
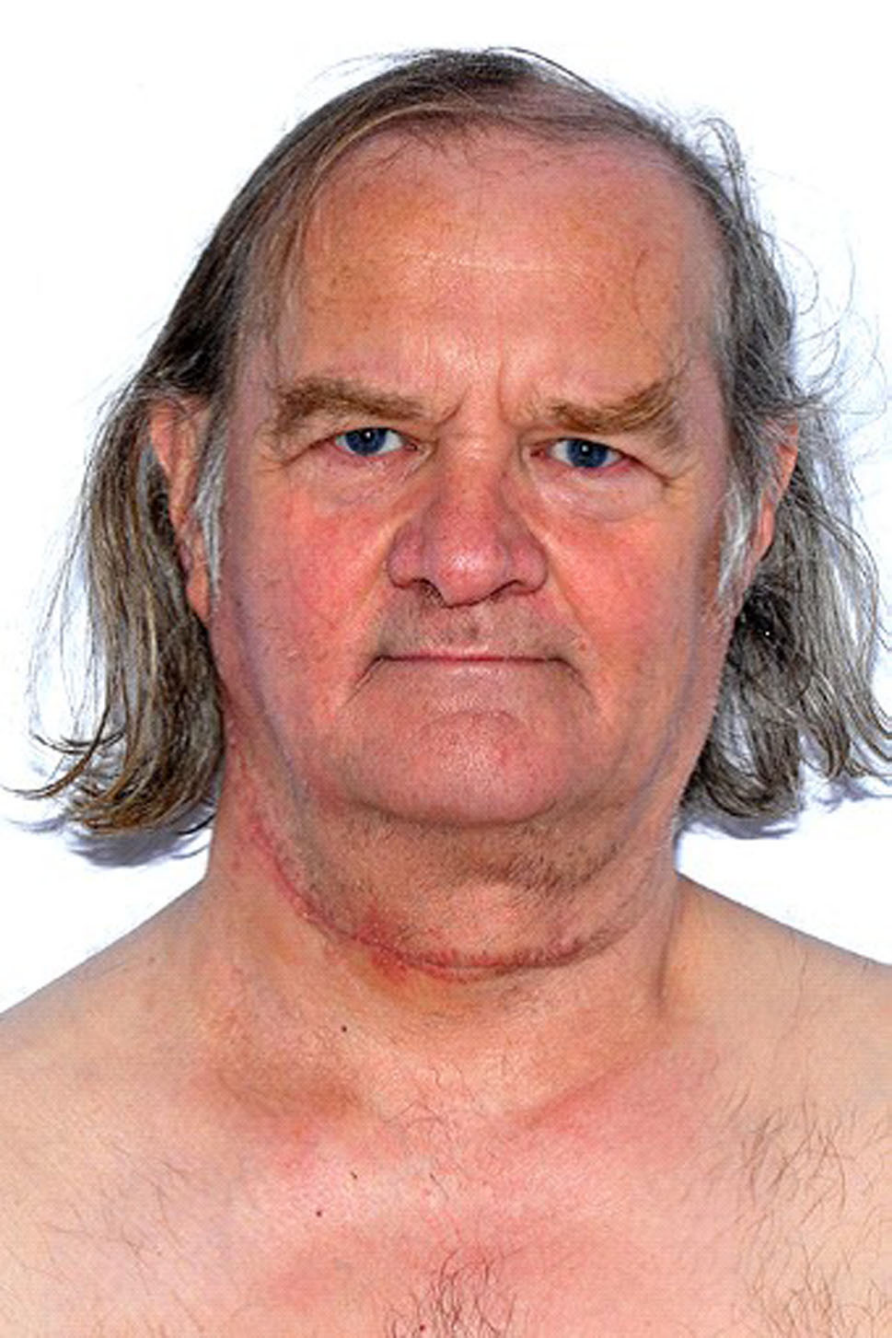
Photograph of patient, post-operation

A 55 year-old gentleman presented to the Ear, Nose and Throat (ENT) clinic with a large neck mass, which had been present for 24 years and had gradually grown in size over time. Despite its significant size, the patient had not sought medical attention for it until then– it never caused discomfort and it did not impede him from performing any activities of daily living. He denied feelings of embarrassment or self-consciousness regarding his appearance and cited being unable to take time off work from his (at the time, new) job, as the main reason for not presenting earlier. The patient described the tumour as “part of him,” and only decided to seek medical intervention after retiring from work. The tumour was on the right side of the neck; firm, mobile and non-tender on palpation. The remainder of the ENT examination (including laryngoscopy) was unremarkable. Right facial nerve function was assessed, and no abnormality was found (Figs. 1, 2). The patient had no other past medical or family history.

**Fig. 3:**
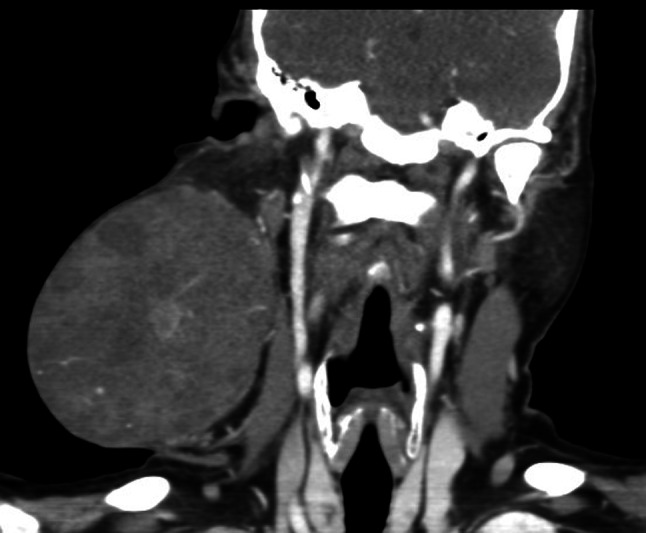
Coronal CT neck - showing tumour pre-operation

**Fig. 4:**
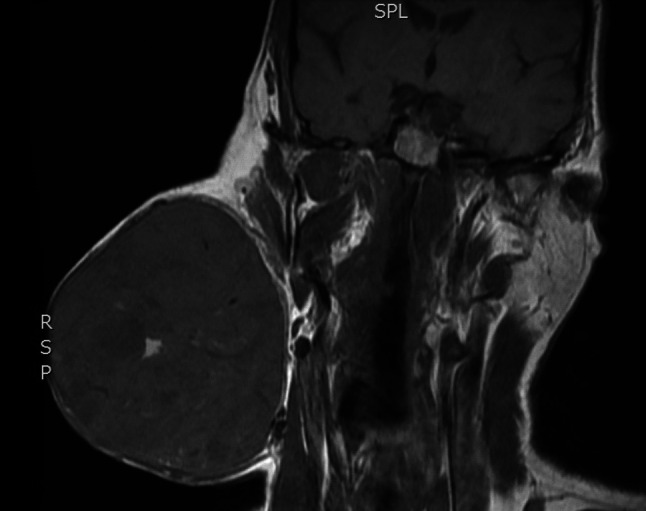
Coronal MRI neck - showing tumour pre-operation

Fine needle aspiration cytology (FNAC) was suggestive of pleomorphic adenoma. Magnetic resonance imaging (MRI) (Fig. 3) and axial and coronal computed tomography (CT) revealed a large right neck mass that measured approximately 12.5 cm in AP dimension, 11 cm in transverse dimension and 11.5 cm in cranio-caudal dimension (Fig. 4). It was mainly solid and showed heterogeneous enhancement. An intensely enhancing focus was noted in its inferior and medial aspect, measuring 22 mm. It was reported to, most likely, arise from the tail of the right parotid gland. The pleomorphic adenoma was not invading into the sternocleidomastoid muscle, carotid artery, internal jugular vein, right submandibular salivary gland or the mandible.

**Fig. 5:**
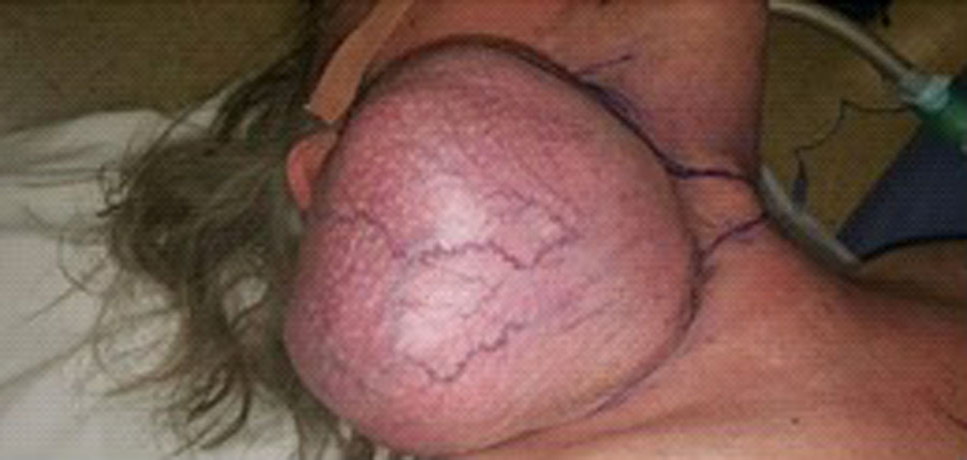
Photograph of patient’s neck pre-operation

**Fig. 6:**
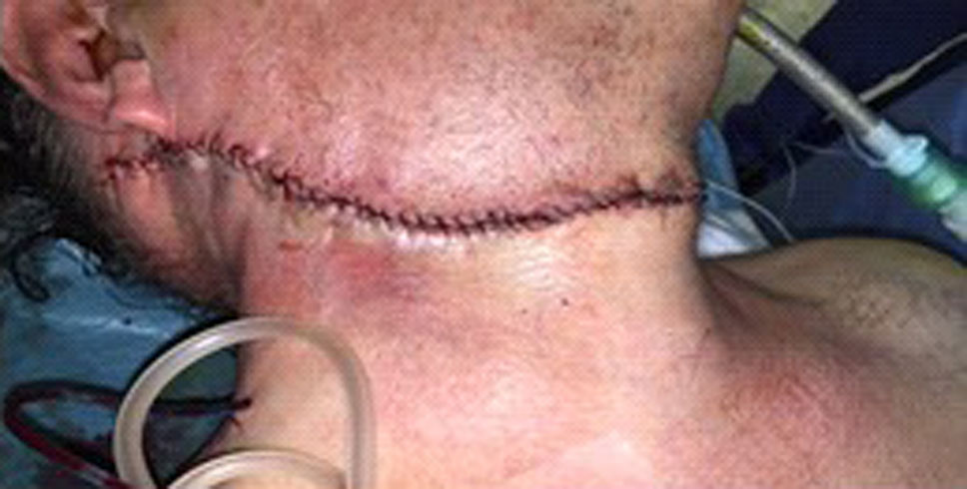
Photograph of patient’s neck post-operation

**Fig. 7:**
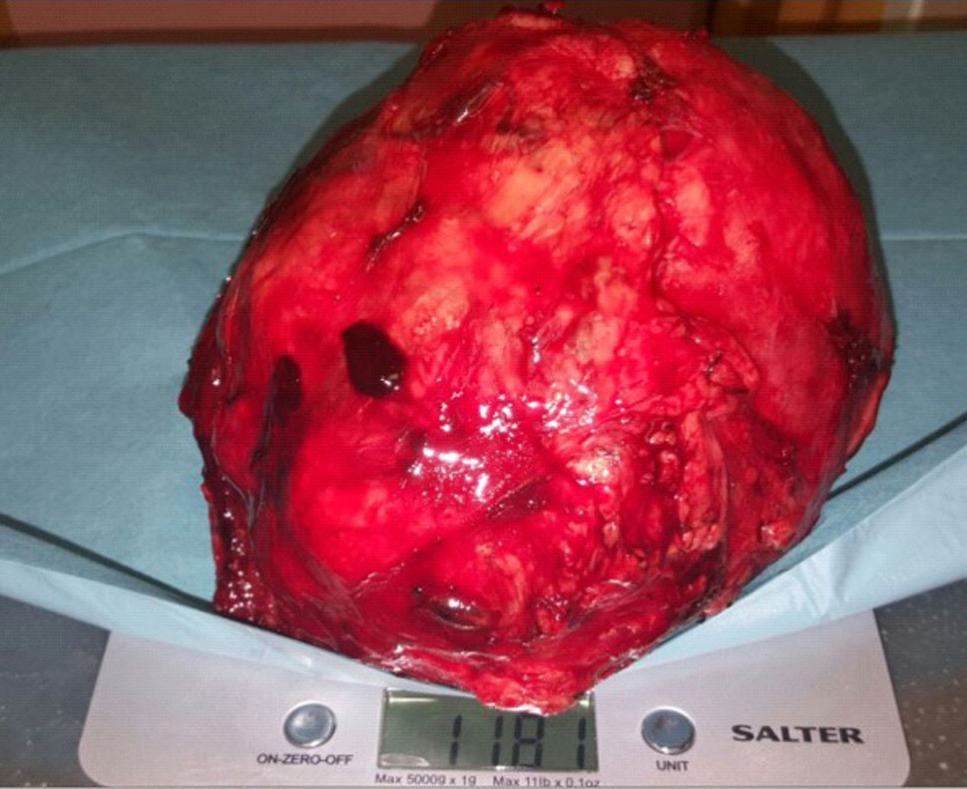
Excised tumour, displaying a weight of 1.181 kg

Due to the size and location of the tumour, a standard parotidectomy incision was not needed. The tumour was excised through a modified skin incision by extracapsular dissection [Figs. 5,6]. The tumour was carefully dissected in a well-defined plane, excised completely and sent for histopathological examination. This occurred under the care of the ENT and Maxillofacial team. The mass, when excised, revealed an exceptionally large tumour measuring 13.5 × 11x11 cm and 1.2 kg [Fig. 7]. A neck drain was inserted. The day after the operation, a mild right marginal mandibular nerve weakness was noticed which, on review two months later, had completely recovered. Post-operatively the patient stayed for 2 days, after which the neck drain was removed and the patient was discharged. The patient undergoes annual follow up and has showed no signs of recurrence almost 5 years later, with no adverse or unanticipated events. His right facial nerve function remains normal.

Histological examination determined it to be a pleomorphic adenoma of the right parotid gland with a small focus of non-invasive carcinoma (carcinoma-in situ). It showed nodules consisting of myoepithelialductal and plasmacytoid cells arranged in a fibrous myxoid or myxochondroid matrix. There was some fibrosis seen as well.

## Discussion

Approximately 75–85% of all pleomorphic adenomas occur in the parotid gland, with only 8% arising in the submandibular gland. Those occupying the minor salivary glands represent 7–15% of all reported cases. Pleomorphic adenomas occur among all age groups, with the incidence rate of about 3.5/100,000. Although initially they are benign, pleomorphic adenomas have the potential to undergo malignant transformation into “carcinoma ex pleomorphic adenomas.” This risk increases with time—it is 1.5% in the first 5 years, after which there is a 9.5% chance to undergo malignant transformation in 15 years [2]. The reported overall prevalence of malignant transformation of pleomorphic adenomas in the literature varies from 3 to 15%, occurring most commonly in the 6th and 7th decades of life [3].

Pleomorphic adenomas are characterised by proliferation of parenchymatous glandular cell- epithelial tissue, mixed with mucoid, chondroid or myxoid tissues. It is a mixed salivary gland tumour of mesenchymal, myoepithelial and duct reserve cell origin. For masses arising from major or minor salivary glands, CT and MRI scans are the gold standard radiological investigations of choice [4–6]. The largest pleomorphic adenoma on record measures 34 × 20 × 26 cm and 8.1 kg, in a female who was 75 years of age [7].

Surgery is the gold standard treatment—the standard procedure for benign tumours is superficial parotidectomy with adequate resection of the margins (simple enucleation is believed to lead to a high local recurrence rate). Care must be taken to preserve the facial nerve. Early surgical intervention is recommended to reduce the risk of malignant transformation, however the literature suggests that in practice, this varies–one retrospective study of 22 carcinoma ex pleomorphic adenoma patients reported a range lead time (evidence of a parotid mass) of 0.1–48 years [4].

## Conclusion

Pleomorphic adenomas have a tendency for local recurrence and some cases undergo malignant transformation. However, in our case, the pleomorphic adenoma was mainly in the neck—attached to just the tail of the parotid gland. On careful review of the CT & MRI scans, one can appreciate a clear plane around the tumour even near the tail of the parotid. As this tumour was mainly in the neck, we excised it with an extra capsular dissection around the tumour in the well-defined plane, using a modified neck incision as shown [Fig. 6]. We used this approach to avoid damage to the main branches of the facial nerve. It is interesting to note the significant length of time the patient lived with this tumour before presenting (24 years). While patients may not experience reduced quality of life, earlier intervention in patients such as these is important to reduce the risk of malignant transformation of the tumour. It is also easier to surgically manage these tumours when they are small, unlike in this case where the. patient allowed it to grow to extreme proportions.

## Data Availability

Data sharing is not applicable to this article as no datasets were generated or analysed during the current study.

## List of Abbreviations

ENT: Ear, nose and throat
FNAC: Fine needle aspiration cytology
AP: Anteroposterior
CT: Computed tomography
MRI: Magnetic resonance imaging
